# Expiratory aerosol pH is determined by indoor room trace gases and particle size

**DOI:** 10.1073/pnas.2212140119

**Published:** 2022-08-29

**Authors:** Liviana K. Klein, Beiping Luo, Nir Bluvshtein, Ulrich K. Krieger, Aline Schaub, Irina Glas, Shannon C. David, Kalliopi Violaki, Ghislain Motos, Marie O. Pohl, Walter Hugentobler, Athanasios Nenes, Silke Stertz, Thomas Peter, Tamar Kohn

**Affiliations:** ^a^Institute for Atmospheric and Climate Science, ETH Zurich, Zurich 8092, Switzerland;; ^b^Environmental Chemistry Laboratory, School of Architecture, Civil and Environmental Engineering, École Polytechnique Fédérale de Lausanne (EPFL), Lausanne 1015, Switzerland;; ^c^Institute of Medical Virology, University of Zurich, Zurich 8057, Switzerland;; ^d^Laboratory of Atmospheric Processes and their Impacts, School of Architecture, Civil and Environmental Engineering, École Polytechnique Fédérale de Lausanne (EPFL), Lausanne 1015, Switzerland;; ^e^Institute of Chemical Engineering Sciences, Foundation for Research and Technology Hellas, Patras 26504, Greece

The work of ref. 1 draws attention to the important role of pH in the inactivation of severe acute respiratory syndrome coronavirus 2 (SARS-CoV-2) in aerosol particles. After very collegial and helpful discussions with the authors of ref. 1, we would like to express our appreciation of their work but also raise a concern about the possible overgeneralization of the alkaline pH found in their study. The pH of exhaled liquid aerosol particles is determined by complex interactions between the particle and the gas phase and is highly dependent on particle size. In our recent work (2), we argue that exhaled aerosol particles become acidic upon equilibration with indoor air.

**Fig. 1.**
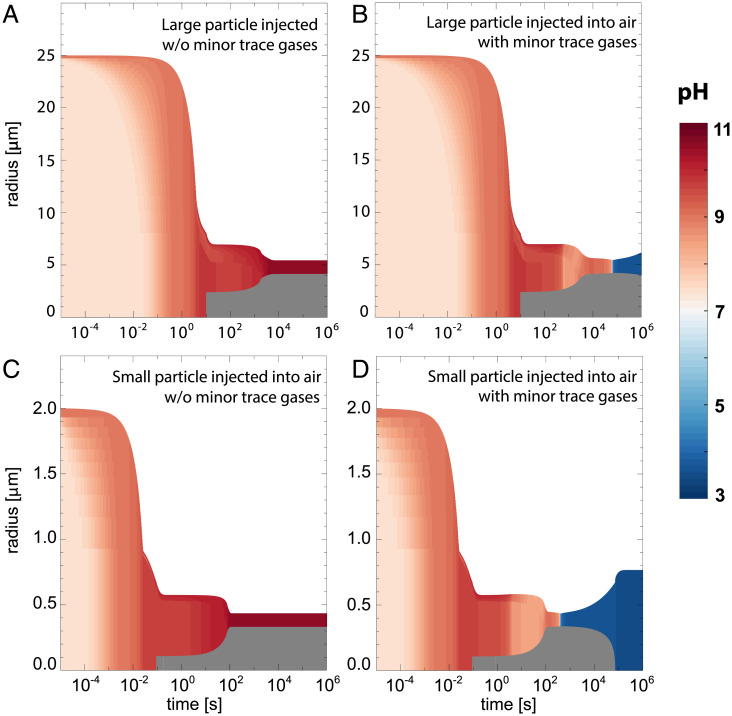
The pH evolution of simulated MEM particles with initial radii 25 μm (*A* and *B*) and 2 μm (*C* and *D*), at *t* = 0 in equilibrium with 99.4% relative humidity (RH) and 5% CO_2_, subsequently injected into air with 50% RH and 400 ppm CO_2_. (*A* and *C*) Laboratory air without minor trace gases (HNO_3_, NH_3_, HCl, CH_3_COOH), and (*B* and *D*) typical indoor air containing trace gases. Colors indicate the pH inside the MEM droplets as a function of time. Dark gray regions are effloresced NaCl. At *t* = 0, pH is 7.4 as in the experiments of ref. 1, with particle composition 0.19158 m (molal) Na^+^, 0.17383 m Cl^–^, 0.0057 m nonsodium and 0.0037 m nonchloride ions, 0.0168 m organics, and Na^+^/(CO_2,aq_ + HCO_3_^–^ + CO_3_^2–^) = 9.15. Assumed trace gas concentrations of indoor air are 0.27 ppb HNO_3_, 36 ppb NH_3_, 0.23 ppb HCl, and 46.7 ppb CH_3_COOH (3).

To understand this apparent contradiction, we simulated the experiments of ref. 1 with our comprehensive microphysical model for exhaled particles developed in ref. 2. We find that our model reproduces the results of ref. 1 very well (Fig. 1*A*). Under their specific experimental conditions—namely, large droplets (*r*_0_ = 25 μm) and pretreated laboratory air—the model produces strongly alkaline conditions (pH > 10). Ref. 1 exposed their particles to a humidity- and temperature-controlled airflow originating from a compressed air supply. The compressed laboratory air is most likely depleted of many of the stickier trace components, such as nitric acid (HNO_3_) and ammonia (NH_3_), due to wall absorption effects. Under these conditions, the model predicts that the particles will become strongly alkaline (pH ≈ 10) after ∼20 min. When, instead, assuming typical indoor air, which contains trace amounts of HNO_3_, NH_3_, and others, the particle pH initially still becomes more alkaline, but eventually acidifies (Fig. 1*B*). In such a large particle, however, gas-to-particle mass transfer kinetics is slow enough so that about a day is required for sufficient amounts of HNO_3_ to condense and make the particle acidic. In contrast, smaller particles (*r*_0_ = 2 μm) become acidic after about 5 min under typical indoor conditions (Fig. 1*D*), while submicron particles require seconds. Thus, not only outdoor aerosol but also exhaled aerosol particles become acidic due to interaction with indoor air, contrary to the statements of ref. 1. Acidification is even more rapid if synthetic lung fluid or mucus derived from primary airway cultures (2) is used instead of the cell culture minimal essential medium (MEM) applied by ref. 1, as the former contains less bicarbonate buffer.

The above underlines that, in addition to CO_2_, other acids and bases present in the air fundamentally shape the pH value of exhaled particles. This can give rise to alkaline or acidic particles, and particle size determines the time required to do so. Large particles take hours to become acidic, while small expiratory particles become acidic in a matter of minutes or less. Finally, it should be noted that particles with an initial radius of 25 μm are removed from room air by gravitational settling within 2 min, while 2-μm particles require about 1 h. Given the above, the smaller particles and their acidity levels may be an important factor that impacts airborne transmission of viruses.
